# Sustainable power solutions for next-generation medical devices

**DOI:** 10.1016/j.mtbio.2025.102055

**Published:** 2025-07-04

**Authors:** Ye Liang, Chi Zhang, Rubing Lin, Junqing Lin, Jishizhan Chen

**Affiliations:** a Centre for Regenerative Medicine and Health, Hong Kong Institute of Science & Innovation, Chinese Academy of Sciences, Hong Kong Special Administrative Region of China; b Hainan Medical University, No.3 Xueyuan Road, Longhua District, Haikou City, 571199, Hainan Province, China; c Department of Orthopedics, Shenzhen Children's Hospital, Shenzhen, Guangdong, China; d Department of Orthopedics, Shanghai Sixth People's Hospital Affiliated with Shanghai Jiao Tong University School of Medicine, 600 Yishan Road, Shanghai, 200233, China; e UCL Mechanical Engineering, Torrington Place, University College London, London, WC1E 7JE, UK

## Abstract

Next-generation medical devices include implantable medical devices (IMDs) and wearables, exemplified by devices such as pacemakers for heart regulation and deep brain stimulators for neurological disorders, which have significantly advanced healthcare by offering critical treatments and improving patient outcomes. However, conventional battery technologies for these devices remain prevalent, and their constraints on longevity, size, and necessity for periodic replacement or recharging pose significant challenges, especially in implantable scenarios, presenting concerns regarding patient safety, healthcare costs, and device reliability. To address these issues, this review investigates alternative energy sources that tap into the intrinsic energy of the human body and delves into a range of promising energy harvesting techniques, including electromagnetic energy harvesting, ultrasound wireless power transfer (US-WPT), energy generation from tissue motion and heartbeats, utilization of body thermal gradients through thermoelectric generators (TEGs), and glucose oxidation within biofuel cells. Each technique is evaluated for its potential to provide a sustainable power source for IMDs and wearables, highlighting distinctive advantages such as dual functionality, enhanced penetration capabilities, access to inexhaustible energy reservoirs from bodily movements, and the biochemical conversion of glucose into electrical energy. Despite their promise, this review also discusses the remaining challenges, future directions, and exciting opportunities associated with these cutting-edge energy harvesting methods, emphasizing the need for multidisciplinary research to overcome current hurdles and unlock new possibilities for self-sustained medical and wearable devices. This review uniquely evaluates energy harvesting techniques through the lens of 'functionally cooperating systems'—emphasizing how synergistic integration of smart materials, adaptive algorithms, and physiological interfaces can overcome fundamental trade-offs between biocompatibility, power density, and clinical viability.

## Graphical abstract

**Figure:**
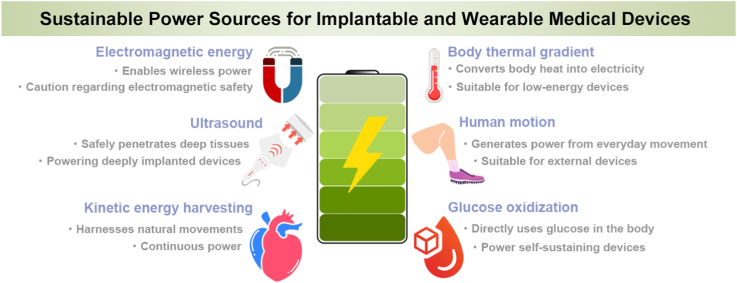

## 1 Introduction

The advancement of next-generation medical devices is accelerating rapidly, driven by the global growth of implantable medical devices (IMDs) and wearable technologies. As of 2023, the IMD market was valued at 26.4 billion, with projections to reach 46.5 billion by 2030, growing at a compound annual growth rate (CAGR) of 8.2 % according to Grand View Research. Wearables, meanwhile, dominated the consumer health tech sector, reaching 115 billion in 2023, and are expected to surpass 265 billion by 2030 according to data at Statista in 2023. This surge is fueled by aging populations, rising chronic disease prevalence, and demand for continuous health monitoring—particularly after the COVID-19 pandemic, which saw remote patient monitoring devices grow by 25 % annually between 2020 and 2023.

IMDs are intricately designed to be inserted inside the human body, either permanently or for extended periods, to monitor, diagnose, or treat various medical conditions. The emergence of IMDs represents a remarkable advancement in the convergence of healthcare and personal electronics [1,2]. Noteworthy examples include implantable cardioverter defibrillators (ICDs), which play a vital role in monitoring heart rhythms and administering corrective shocks to mitigate life-threatening arrhythmias. Additionally, artificial heart valve devices are implemented to replace damaged or diseased heart valves, ensuring proper blood circulation through the heart. Deep brain stimulators (DSSs) stand out as another remarkable innovation utilized in treating neurological disorders such as Parkinson's disease by delivering targeted electrical stimulation to specific brain regions. These groundbreaking innovations hold immense potential in reshaping continuous health monitoring and therapeutic interventions, ultimately enhancing the quality of life for individuals managing chronic conditions [3,4]. Achieving this integration of advanced technological components with human physiology is crucial in this rapidly evolving field. Miniaturization, biocompatibility, and sustainable power solutions that address the limitations of traditional batteries, such as lifespan, replacement frequency, size, and environmental impact, are needed [5].

**Fig. 1:**
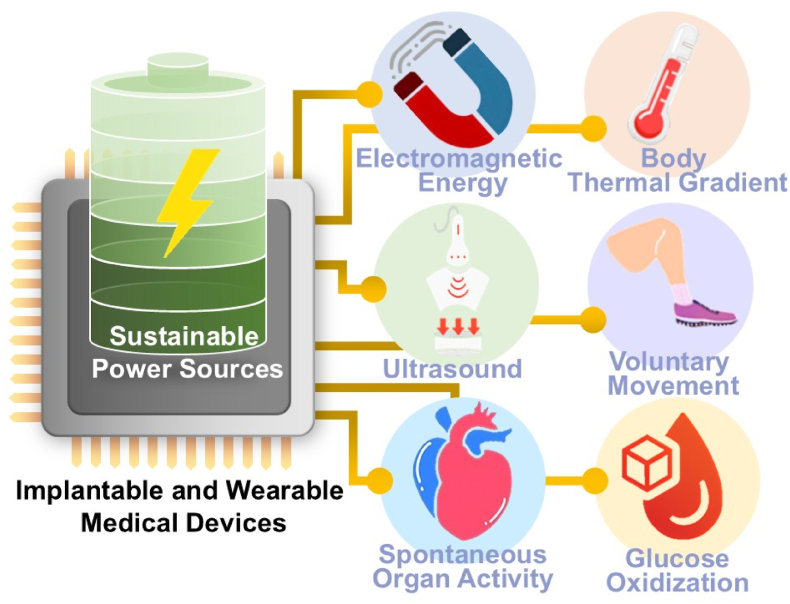
Representative sustainable power sources for IMDs and wearable technologies.

To date, several promising energy harvesting methodologies have emerged as viable power sources for both IMDs and wearable technologies [6] (Fig. 1). Specifically, approaches such as electromagnetic energy harvesting and ultrasound wireless power transfer (US-WPT) rely on external power sources. Electromagnetic energy harvesting offers dual benefits by enabling simultaneous power transmission and data exchange, making it ideal for devices such as smart contact lenses that require continuous functionality and information sharing. For example, smart contact lenses equipped with sensors for glucose monitoring can benefit from this technology by maintaining continuous operation without the need for frequent battery replacements. US-WPT provides an effective alternative capable of penetrating electrically conductive materials without electromagnetic interference, making it suitable for devices implanted deeper within the body, such as cochlear implants [[7], [8], [9]].

In contrast, other approaches take advantage of human physiology to generate power. Spontaneous organ activity, for example, uses tissue movements and heartbeats to create a virtually unlimited power source suitable for pacemakers. This method leverages the body's natural, continuous movements to generate electricity, potentially eliminating the need for battery replacements in life-saving devices. TEGs utilize body thermal gradients to convert body heat into electrical power via the Seebeck effect. This technology can be applied to wearable devices such as fitness trackers, which can harness the heat generated by the body during physical activity to extend their operational life. Additionally, biochemical reactions in glucose-based fuel cells transform the chemical energy of glucose into electricity, offering sustainable options for powering IMDs and wearable electronics. For example, biofuel cells implanted in diabetic patients can use glucose from the bloodstream to power continuous glucose monitors, providing a self-sustaining energy source.

Despite their potential, these energy harvesting methods face various challenges that must be addressed to realize their full potential. Key issues include weak coupling, conversion efficiency, biocompatibility, and long-term effects on human health due to prolonged exposure to energy harvesting methods. For example, the efficiency of energy conversion in thermoelectric generators is currently limited, and improving this efficiency is crucial for their practical application. Similarly, ensuring the biocompatibility of materials used in biofuel cells is essential to prevent adverse immune responses.

This article delves into detailed discussions of each energy harvesting technique, outlining their mechanisms, advantages, and obstacles hindering widespread adoption. By investigating these factors, we aim to set the stage for a critical analysis of current energy harvesting technologies applicable to IMDs and wearable devices. Through this comprehensive exploration, we hope to identify pathways for future research and development that could pave the way for more sustainable, efficient, and user-friendly power sources. Unlike prior reviews, we critically dissect cross-cutting challenges: (i) the biocompatibility-power density paradox, (ii) dynamic coupling efficiency in physiological environments, and (iii) AI-driven energy management. Our analysis reveals that hybrid systems leveraging spontaneous organ activity and voluntary motion show the highest potential for clinical translation.

## 2 Development of sustainable power sources

The concept of sustainable power for medical devices emerged from the recognition of the limitations associated with traditional batteries, including their finite lifetime, the need for replacement or recharging, and potential risks associated with battery leakage or failure. Early research in this field focused on exploring alternative energy sources that could harness energy from ambient sources such as mechanical motion, body heat, and biochemical reactions. Key early works in this area include the development of piezoelectric energy harvesters, which convert mechanical strain into electrical energy [10]. Additionally, TEGs that utilize temperature gradients to produce electricity have also been explored [11]. These initial studies laid the groundwork for the development of sustainable power strategies by demonstrating the feasibility of harvesting energy from the human body.

A significant breakthrough in the development of sustainable power strategies came with advancements in materials science. Researchers have begun to explore new materials with enhanced properties that could improve the efficiency and reliability of energy harvesters. For example, the discovery of piezoelectric materials with high piezoelectric coefficients, such as lead zirconate titanate (PZT) and barium titanate (BaTiO_3_), led to the development of more efficient piezoelectric energy harvesters [12]. Similarly, the development of high-performance thermoelectric materials, such as bismuth telluride (Bi_2_Te_3_) and silicon germanium (SiGe), has improved the efficiency of TEGs [13]. These material advancements enabled the development of prototypes that demonstrated the practical application of sustainable power strategies. For example, researchers have developed wearable piezoelectric energy harvesters that can power small electronic devices by converting the mechanical energy from walking [14]. Additionally, TEGs have been integrated into implants to harvest energy from body heat [15].

With the development of prototypes, the next challenge was to integrate sustainable power systems with medical devices. This requires overcoming technical barriers such as compatibility with existing device architectures, ensuring sufficient power output, and maintaining biocompatibility. Researchers have worked closely with medical device manufacturers to develop customized solutions that can be seamlessly integrated into devices such as pacemakers, neural implants, and wearable health monitors. For example, researchers developed a piezoelectric energy harvester that was integrated into a pacemaker lead, enabling the device to be powered by the mechanical energy from heartbeats [16].

As sustainable power strategies began to be implemented in real-world applications, researchers focused on optimizing and refining the systems to improve their performance and reliability. This included enhancing energy-harvesting efficiency, reducing power consumption, and improving the durability of the components. For example, researchers have developed adaptive algorithms that can optimize the performance of energy harvesters on real-time environmental conditions [17]. Additionally, efforts have been made to reduce the power consumption of medical devices by implementing low-power electronics and energy-efficient communication protocols [18].

In recent years, new technologies that further enhance the capabilities of sustainable power strategies for medical devices have emerged. For example, triboelectric nanogenerators (TENGs) have been developed that can harvest energy from friction and contact electrification [19]. Additionally, wireless power transfer systems, such as inductive coupling and resonant magnetic coupling, have been explored as a means to provide continuous power to implants without the need for batteries [20].

## 3 Different types of sustainable power sources for IMDs and wearable technologies

Given the ever-evolving landscape of energy harvesting methodologies, the quest for novel approaches to power implantable medical devices and wearable technologies has led to the emergence of cutting-edge techniques. These innovative methods, which are designed to extract energy from both the human body and its ambient environment, hold the promise of sustainable power generation, a continuous energy supply, and heightened efficiency. We explore the latest developments in sustainable power sources for IMDs, focusing on electromagnetic energy, ultrasound, tissue motion and heartbeat, body thermal gradient, glucose oxidization and voluntary motion. As outlined in Table 1 below, the exploration of these new energy harvesting techniques represents a pivotal step toward reshaping the future of healthcare technology.

**Table 1:** Different power strategies for IMDs and wearable technologies.

Energy Harvesting Technique	Mechanism	Advantages	Obstacles
Electromagnetic Energy Harvesting	Convert ambient electromagnetic radiation into electrical energy	Relatively constant power source, scalability, device integration	Limited power output, dependence on ambient sources, efficient rectification
Ultrasound Wireless Power Transfer (US-WPT)	Use ultrasonic waves for wireless power transfer	Safe, efficient power transfer, penetrates tissues and materials	Long-distance power transfer efficiency, tissue heating, alignment requirements
Spontaneous organ activity	Capture mechanical energy from tissue movements and heartbeats	Abundant energy source from body movements, suitable for active regions	Low power density, efficient energy conversion, activity level variability
Thermoelectric Generators (TEGs)	Utilize temperature differentials for electricity generation	Continuous power from body heat, sustainable energy source	Limited efficiency in heat-to-electricity conversion, thermal management, bulkiness
Voluntary movement	Harness kinetic energy from human movement	Utilizes everyday motion for power generation, potential for continuous energy supply	Efficiency in energy conversion, variability in activity levels, device integration
Glucose-based Biofuel Cells	Extract electrical energy from glucose oxidation	Long-term power generation, sustainable without external charging	Biocompatibility, stability of glucose oxidation, glucose supply

### 3.1 Electromagnetic energy

**Fig. 2:**
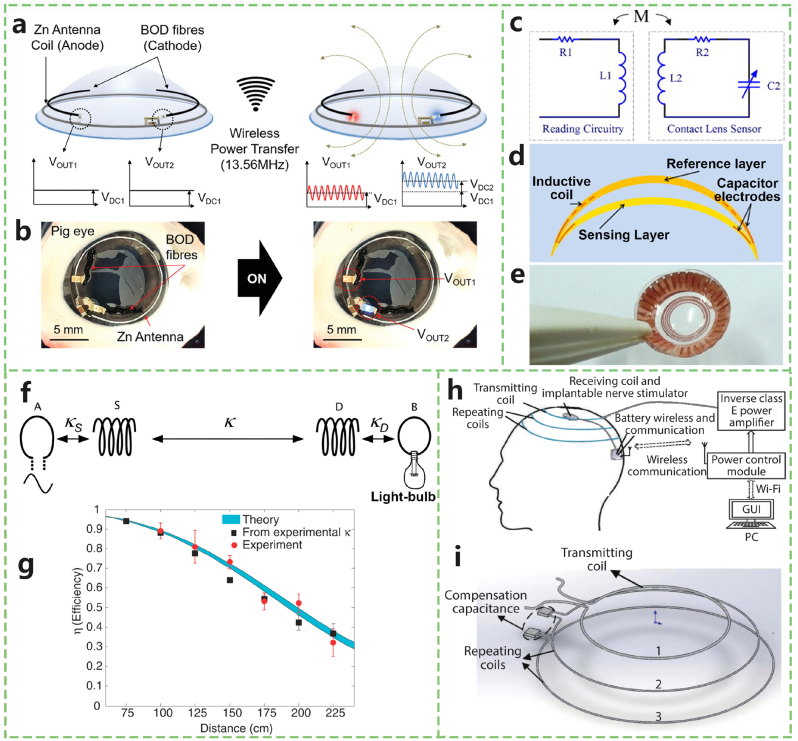
Implantable devices powered by electromagnetic energy. a) Schematic of a hybrid wireless power transfer and Zn–air battery system integrated into a smart contact lens. b) Experimental image of the smart contact lens mounted on a pig eye, where red and blue LEDs are illuminated under a 13.56 MHz AC wireless power supply. c) Equivalent circuit diagram showing inductively coupled LC resonators between a contact lens sensor and an external reader. d) Cross-sectional view of a smart contact lens embedding the LC sensing element within a double-layer silicone rubber structure. e) Gross image of a contact lens sensor incorporating an electrode and inductive coil. f) Schematic of a MCR wireless power transfer system using two self-resonant coils. g) Experimental and theoretical comparison of WPT efficiency as a function of coil separation distance. h) Schematic of a wearable WPT and wireless communication platform based on the MCR approach. i) Structural arrangement of the transmitting and repeating coils used to extend the power transfer range. Adapted from Refs. [20,21,23,24]. (For interpretation of the references to colour in this figure legend, the reader is referred to the Web version of this article.)

Owing to the significant influence of the dielectric properties of biological tissues on electric fields, magnetic fields are favoured for power transfer in IMDs. Electromagnetic (EM) wireless transfer systems have gained considerable attention because of their intrinsic energy transfer mechanisms. A notable advantage of this method is its ability to support continuous data monitoring and exchange with an external reader, making it ideal for applications such as intraocular pressure [21] (Fig. 2a and b) and glucose measurement from tears [22]. This method has been particularly effective in powering smart contact lenses.

Several approaches to wireless power transfer (WPT) for IMDs exist, including inductive coupling (IC), far-field radio frequency (RF), magnetically coupled resonant (MCR), and mid-field WPT [25]. Takamatsu et al. [23] developed a hybrid power system that combines a WPT system with a bioabsorbable metal‒air primary battery for smart contact lenses (Fig. 2c–e). This device delivers multifunctional direct current (DC) and alternating current (AC) outputs, generating DC power through a zinc loop anode and a bilirubin oxidase biocathode in artificial tears. The zinc-based loop anode also functions as an antenna to receive a resonant frequency of 13.56 MHz. This system can produce sufficient voltage (>1.5 V) to operate red and blue LEDs. Zada et al. [26] designed a smart contact lens with a self-tuned spiral-shaped antenna operating at 920 MHz and 2.4 GHz for WPT and data telemetry. This device achieved a power transfer efficiency (PTE) of −17.85 dB at a 12 mm distance between the transmitter and the antenna, successfully lighting a micro-LED in saline. Kurs et al. [20] initially proposed MCR-WPT, which requires fixed distances between coils to maintain voltage stability (Fig. 2f and g). To address this, Zhang et al. [24] designed a wearable MCR-WPT system with a four-coil structure and closed-loop control to stabilize voltage fluctuations caused by coil displacement, enhancing magnetic coupling and WPT efficiency (Fig. 2h and i).

Mid-field WPT has advantages in delivering power through deep human tissue. A novel compact mid-field WPT system includes an implantable planar inverted-F antenna with a 9 × 13 mm^2^ receiver [27]. Simulation experiments using pork muscle demonstrated that this system could achieve over 5.6 mW of received energy at 1 W of output power at a subwavelength distance.

Significant advancements have been made in innovative technologies for implantable cardiac devices. Zurbuchen et al. [28] developed a generator consisting of a stack of permanent magnets with a flux density of 1.43 T, copper coils, and spring supports. This device converts mechanical energy from cardiac motion into electrical energy through electromagnetic induction. Franzina et al. [29] utilized electromagnetic claw generator technology to create a prototype that converts spontaneous organ activity into electrical energy for pacing circuits. This device features nitinol hooks for secure implantation in the endocardium, ensuring efficient energy conversion. Zurbuchen et al. [30] proposed a method for harvesting energy from endocardial heart motions. They developed a generator based on electromagnetic induction that converts the spontaneous organ activity from heart movements into electrical energy. The device is equipped with serially aligned copper coils and a linear arrangement of permanent magnets suspended in springs. The spring design was optimized via finite element analysis to ensure effective oscillation and energy harvesting during heart motion. The final design was validated through both bench and in vivo tests, which demonstrated its ability to generate electrical power at various heart rates. This approach could serve as an effective alternative for extending the lifespan of cardiac implantable electronic devices and has the potential to transform pacemakers into battery-free and leadless systems, addressing major limitations of current technologies.

Despite their advantages, EM field usage in WPT faces challenges such as weak coupling in IC systems when encountering misalignment or large transmission distances [25]. Frequent eye movements, for example, can affect the stability of PTE in smart contact lenses. Continuous EM field exposure may also pose health risks, including nerve stimulation and a rise in temperature [31]. To mitigate these risks, safety standards such as the specific absorption rate (SAR) for frequencies between 100 kHz and 300 GHz have been established by the International Commission on Non-Ionizing Radiation Protection (ICNIRP) [31]. According to IEEE Standard C95.1 [32], the maximum SAR value must remain below 1.6 W/kg for any 1 g of body tissue and below 0.08 W/kg for the whole body, with higher limits for extremities. Ensuring compliance with these restrictions is essential for the safe use of EMF in medical devices.

Shi's group has pioneered the development of mini-generators that harness various low-grade energy sources such as bubbles, winter sunshine, and blood pressure differentials to produce useable electricity[]. These mini-generators operate on the principle of 'functionally cooperating systems', incorporating smart materials that work in sequence to perform complex tasks like energy conversion. Noteworthy is the application of these mini-generators to power pacemakers using blood pressure differentials, showcasing their potential in critical medical contexts. Compared to Triboelectric Nanogenerators (TENG) with high voltage outputs, these electromagnetic mini-generators offer higher induced currents. Challenges, however, persist in miniaturizing these devices and ensuring compatibility with implanted medical devices, highlighting the need for further research and development to optimize their functionality within the constraints of miniaturization and implant compatibility [[33], [34], [35], [36]].

### 3.2 Ultrasound wireless power transfer (US-WPT)

US-WPT transfers energy in the form of sound or vibration waves, which benefits from deeper penetration depths. The basic principle of this method is that an implanted transducer receives focused ultrasound from the emitter and converts the ultrasound energy into electrical energy, which is used for powering IMDs. Compared with electromagnetic energy, ultrasound can travel through electrically conductive materials, and it neither interferes with electromagnetic fields nor harms the human body [37]. In terms of efficiency, ultrasound WPT significantly outperforms inductive coupling approaches and is more suitable for implanted biomedical applications [[38], [39], [40]].

**Fig. 3:**
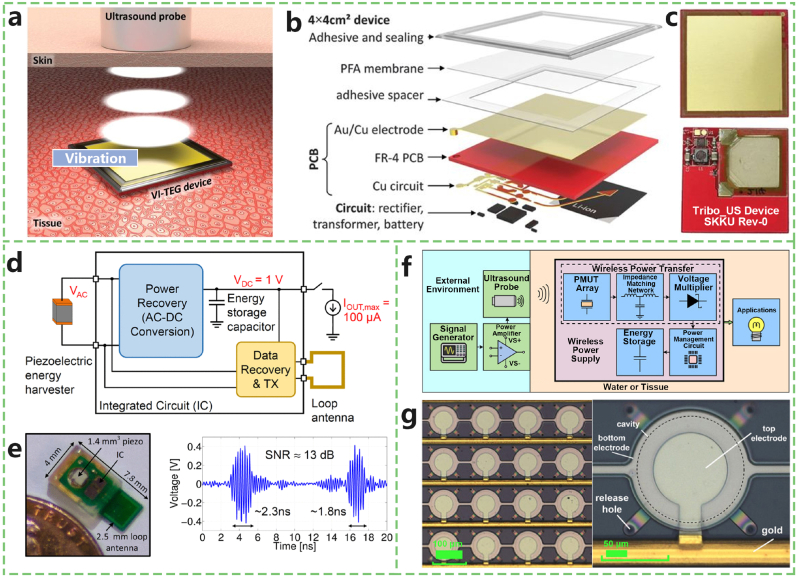
Implantable devices powered by ultrasound. a) Schematic of the ultrasound energy harvesting principle and device using a VI-TEG beneath the skin. b) Exploded view of the internal structure of the VI-TEG. c) Photographs showing the front and back sides of the VI-TEG. d) Block diagram of an implantable device composed of a piezoelectric energy receiver, an integrated circuit chip, and a loop antenna. e) Image of the fully packaged device along with results from an end-to-end blind test, demonstrating successful recovery of ultrawideband pulses at the external receiver. f) Block diagram of another implantable system utilizing ultrasound-induced wireless energy. g) Microscopic (left) and magnified (right) views of the AlN-PMUT array. Adapted from Refs. [[42], [43], [44]].

Several recent advancements in ultrasound-based wireless power transfer technologies have demonstrated their effectiveness and potential. Yokoi et al. [41] proposed a series resonant US-WPT system on the basis of a series resonant tank. This system has a high system power conversion efficiency of approximately 10 % and a maximum output power of 260 mW. Charthad et al. [42] used merely 5 % of the FDA acoustic diagnosis limit to generate 100 μW direct current (DC) power via ultrasonic power transfer and a hybrid bidirectional data communication link. The implant measures 4 mm × 7.8 mm and is composed of a piezoelectric receiver, an integrated circuit, and an off-chip antenna (Fig. 3d and e). This prototype has the potential to provide high power levels to implants with mm and submm sizes inside the body up to ∼10 cm.

Zhicong Rong et al. [43] introduced an ultrasound-induced wireless power supply system based on AlN piezoelectric micromachined ultrasonic transducers (PMUTs). The system uses a PMUT array as a passive wireless power receiver and integrates wireless power transfer, power management, and energy storage functions (Fig. 3f and g). The output power density of the wireless receiver reaches 7.36 μW/mm^2^, which is within FDA safety limits for ultrasound power. The system achieves an output power of 18.8 μW, and after power management, it can charge a 100 μF capacitor to 3.19 V, sufficient to power various low-power implantable biomedical devices such as neural electrical stimulators, biosensors, and intrabody communication devices. When implemented on a PCB with a diameter of 1 cm, the wireless power supply system employs an AlN thin film to ensure biocompatibility and CMOS compatibility, paving the way for safer and miniaturized wireless power supply devices in future developments.

Ronan Hinchet et al. [44] demonstrated a thin, implantable vibrational triboelectric generator that effectively harvests ultrasound energy transmitted through the skin and liquids (Fig. 3a–c). This study explored triboelectric technology's ability to capture ultrasound energy in liquids and soft tissues. The prototypes can generate power in the milliwatt range, enabling the recharging of capacitors and lithium-ion batteries. Researchers have observed performance variations among VI-TEG prototypes and noted that better control of membrane thickness, tension, and air gaps could influence vibrations and potentially improve performance. The device generates electrical energy through contact electrification, with ultrasound causing micrometer-scale displacement of a polymer membrane. A charging rate of 166 microcoulombs per second was achieved for a lithium-ion battery in water. In vitro, ultrasound energy transfer resulted in a voltage of 2.4 V and a current of 156 microamps. These findings indicate that capacitive triboelectric technology is a promising contender against piezoelectric technology for in vivo ultrasound energy harvesting to power medical implants.

**Additionally, US-WPT has advantages in size scaling compared with radiofrequency strategies, which is critical for IMDs.** Researchers are working to miniaturize IMDs while maintaining optimal function and performance. Mazzilli et al. [45] displayed miniaturized transducers with sizes of 30 mm × 96 mm and 5 mm × 10 mm, respectively. A system efficiency of 1.6 % was measured at a transmitter‒receiver distance of 105 mm in water. Yi et al. [46] developed a miniaturized 1–3 piezoelectric receiving transducer with a diameter of 3.7 mm for ultrasonic energy conversion. The output power was reported to have reached 60 mW at a distance of 150 mm in water. The following in vitro and in vivo animal experiments revealed that the miniaturized transducer could successfully receive focused ultrasonic energy and convert it to electrical power without obvious damage to animal tissues. This work illustrates the possibility of obtaining greater power with a smaller implanted device when US-WPT is used.

Zeinab Kashani and Mehdi Kiani [47] highlighted that using an external phased array design for ultrasound wireless power transfer enables efficient power delivery to millimeter-scale biomedical implants while ensuring compliance with FDA safety standards through optimized beam focusing and steering. Antonino Proto et al. [48] developed an intrabody system model to investigate the characteristics of ultrasound power transmission. The study employed a Langevin transducer as the transmitter and an AlN-based square piezoelectric micromachined ultrasonic transducer (pMUT) as the receiver. The impedance, phase, velocity, displacement, and acoustic pressure field of the transducers were measured in the experiments, and the system performance, including the voltage and power output, was analysed. With an approximate root mean square voltage input of 35 V, a power density of 21.6 μW cm-2 was achieved, demonstrating effective charging capability for implantable devices.

The major drawback of using ultrasound is that internal skeletons cause obvious energy attenuation; accordingly, the increase in transferred power is limited to the mW class [41]. Additionally, continuous ultrasound delivery leads to a standing wave, and the receiving device should be located at a minimum pressure point. According to FDA regulations, the maximum acoustic power flux of a wireless power supply source must not exceed 720 mW cm^−2^ [[39], [49]].

### 3.3 Energy harvesting from spontaneous organ activity

**Fig. 4:**
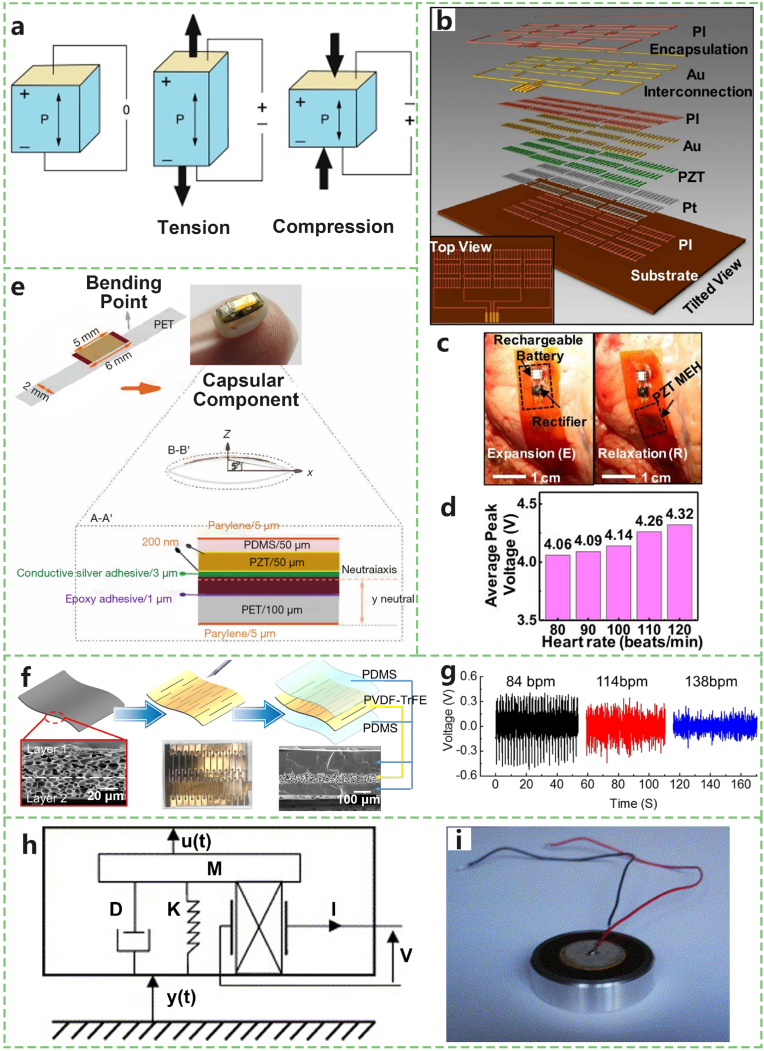
Implantable devices powered by spontaneous organ activity. a) Schematic of the piezoelectric effect, where piezoelectric materials generate electric charge under cyclic tension and compression. b) Exploded-view schematic and top view (inset) of a device based on thin ribbons of PZT, Ti/Pt, and Cr/Au electrodes. c) PZT-based mechanical energy harvester (MEH) integrated with a rectifier and rechargeable battery, mounted on the right ventricle of a bovine heart and shown during expansion (left) and relaxation (right). d) Average peak voltages generated by the PZT MEH at heart rates ranging from 80 to 120 bpm. e) Schematic design and dimensional layout of a flexible piezoelectric energy harvester. f) Fabrication process of a kirigami-inspired lead-based spontaneous organ activity harvester, including thin film preparation, kirigami patterning, and device encapsulation. g) Voltage output profiles collected from a porcine heart under three different heart rates. h) Equivalent mechanical-electrical circuit model of the unimorph diaphragm transducer, with mass (M), spring constant (K), damping (D), mass displacement (u), current (I), and voltage (V). i) Physical structure of the unimorph diaphragm transducer composed of two layers of PZT and a brass substrate (radius = 20.5 mm). Adapted from Refs. [51,52,53,54,55].

Harvesting power directly from natural tissue motion represents an attractive alternative for IMDs. The nonstop regular motion, such as eye blink, contractile and relaxation motions of the heart, lung, and diaphragm, provides an inexhaustible source of mechanical power throughout the life cycle. These intrinsic motions require little effort and consciousness to accomplish, which can largely improve the comfort of users and avoid inconvenient daily charges. Piezoelectric materials allow conversion between mechanical and electric power (Fig. 4a), which offers routes to energy harvesting in such cases. This is because mechanical deformation can lead to electrical charge accumulation in certain solid materials [50].

Several innovative approaches have been explored to harness mechanical energy from various bodily movements. Dagdeviren et al. [51] demonstrated an integrated system that harvests energy from heartbeat and lung movements. The key functional element is a capacitor-type structure consisting of multiple layers of PZT, Ti/Pt, and Cr/Au electrodes (Fig. 4b). The devices were tested in vivo on the hearts and lungs of various species. In ovine or bovine hearts, the voltage output reached a range of 2–4 V, and both the voltage and frequency could be further increased by increasing the force and degree of contractility (Fig. 4c and d). Comparable results were obtained from ovine and bovine lungs and porcine hearts, with an efficiency of ∼2 %. This prototype validates the use of organ motions to power pacemakers without or with less battery assistance. Xie et al. [52] explored the conversion of spontaneous organ activity from the heart into electrical energy through an implanted piezoelectric energy collector (Fig. 4e). Their study revealed that such a device could successfully power a cardiac pacemaker, providing self-powered pacing by transforming this energy into electrical pulses that stimulate myocardial tissue. The in vivo experiments conducted showed promising results for a sustainable pacing solution without the need for external energy sources. Lu et al. [56] focused on utilizing ultraflexible piezoelectric devices that integrate seamlessly with the heart. These devices aim to harvest biomechanical energy from heart movements. The key feature of this approach is the ultraflexibility of piezoelectric devices, which allows for efficient energy capture without interfering with the natural function of the heart or other organs. This strategy could revolutionize how IMDs are powered, reducing or eliminating the need for battery replacements and thereby enhancing patient comfort and device sustainability. Hwang and colleagues developed a self-powered cardiac pacemaker using a flexible single-crystal PMN-PT piezoelectric energy harvester [57]. Their device exhibited notable efficiency in converting biomechanical energy from the heart into electrical energy, demonstrating a viable path towards the realization of self-sustaining medical implants. The material choice and design considerations in this study underscore the importance of matching the piezoelectric properties with the biomechanical environment of the heart. Vyas and Saxena's work on piezoelectric power generation from human heart vibrations further emphasized the feasibility of tapping into the heart's natural movements for energy [58]. Their research suggested that piezoelectric materials can efficiently capture the vibrational energy generated by the heartbeat, offering a reliable and continuous power source for IMDs. This approach could significantly reduce the reliance on traditional battery-powered devices, moving towards a future where medical implants are powered by the very organs they are designed to assist.

Using flexible and advanced designs, a **novel contractile cardiomyocyte-driven piezoelectric nanofiber (CCDPN) bio generator** [59] utilizes piezoelectric polyvinylidene fluoride (PVDF) nanofibers to harvest mechanical energy directly from the heart. The CCDPN bio generator represents an innovative use of nanomaterials in energy harvesting, offering a potentially highly efficient method for powering IMDs through the natural contraction and relaxation cycles of the heart. Zhe Xu et al. [53] developed a kirigami-inspired energy harvester in their study, which was fabricated from high-quality piezoelectric composite materials (Fig. 4f). This device effectively converts biomechanical energy into electrical energy and seamlessly integrates it into clinically used lead-based pacemakers. They achieved outstanding flexibility and high voltage output through optimization of the kirigami structure (Fig. 4g). The optimization process was validated through finite element analysis and experimental testing, confirming its effectiveness in practical applications. Finally, the device was evaluated through both in vitro and in vivo animal experiments, which demonstrated its ability to generate significant electrical voltage from the biomechanical motions of the heart through a straightforward implantation procedure.

The motions of the heart and lungs can provide a constant source of energy, and the magnitude and stability of this energy are influenced by the physiological state of the organism, such as the activity level and health condition. For example, individuals engaging in physical labor versus those with sedentary office jobs exhibit different heart rates and contraction forces, which could lead to fluctuations in energy harvesting efficiency. Additionally, physiological differences between individuals may affect the universality and reliability of energy harvesting systems. The efficiency of converting mechanical energy to electrical energy via piezoelectric materials is generally limited by the properties of the materials and the optimization of the design. Current research focuses on a few specific piezoelectric materials, such as PZT, which, despite their high efficiency, may pose biocompatibility issues that limit their application in long-term implanted medical devices. Furthermore, the devices require precise design and placement to maximize energy harvesting efficiency. This necessitates that the device operates stably within the complex biological environment while avoiding undue pressure or damage to surrounding tissues.

Advancements in piezoelectric energy harvesting from mechanical vibrations have led to several notable innovations. Minazara et al. [54] utilized the mechanical vibrations of a single-layer piezoelectric circular membrane to generate electrical energy from the diaphragm (Fig. 4h and i). The operational principle involves inducing AC voltage signals in the piezoelectric layer through mechanical vibrations, transmitting these signals via electrode layers, and converting them into DC voltages through rectification to charge and store capacitors. The piezoelectric membrane includes a nonactive bottom layer, typically a thick brass layer designed to increase the bending torque around its motion, and an active piezoelectric material layer (PZT in this study) affixed above the nonactive layer to maximize the strain and electrical output efficiency. Wei Wang et al. [60] demonstrated the method of harvesting electrical energy from mechanical vibrations via a piezoelectric circular membrane array, optimizing the array configuration through series and parallel connections to significantly increase the energy output efficiency. Chaoqun Xu et al. [61] summarized research findings on piezoelectric circular diaphragm (PCD) energy harvesters using a ring-shaped ceramic disk. Their study revealed a nonuniform strain distribution within the PCD, leading to an uneven output voltage and a reduced energy harvesting efficiency. By optimizing the PCD through diameter reduction and center hole incorporation, they successfully enhanced the energy harvester's output power and performance. These studies illustrate the ongoing advancements in piezoelectric energy harvesting technology.

Although the concept of powering devices through tissue motion is theoretically attractive, its practical application needs to address a range of engineering and biomedical challenges. These include the long-term stability of the device, energy storage and management solutions, and compatibility with existing medical devices for integration. Despite these challenges, research into energy harvesting from tissue motion and heartbeats holds significant potential. Future efforts could explore new piezoelectric materials and designs to improve energy conversion efficiency and biocompatibility. Moreover, developing advanced energy management systems to ensure a stable and reliable power supply under varying physiological states will be key to commercializing this technology and broadening its application.

### 3.4 Body thermal gradient

**Fig. 5:**
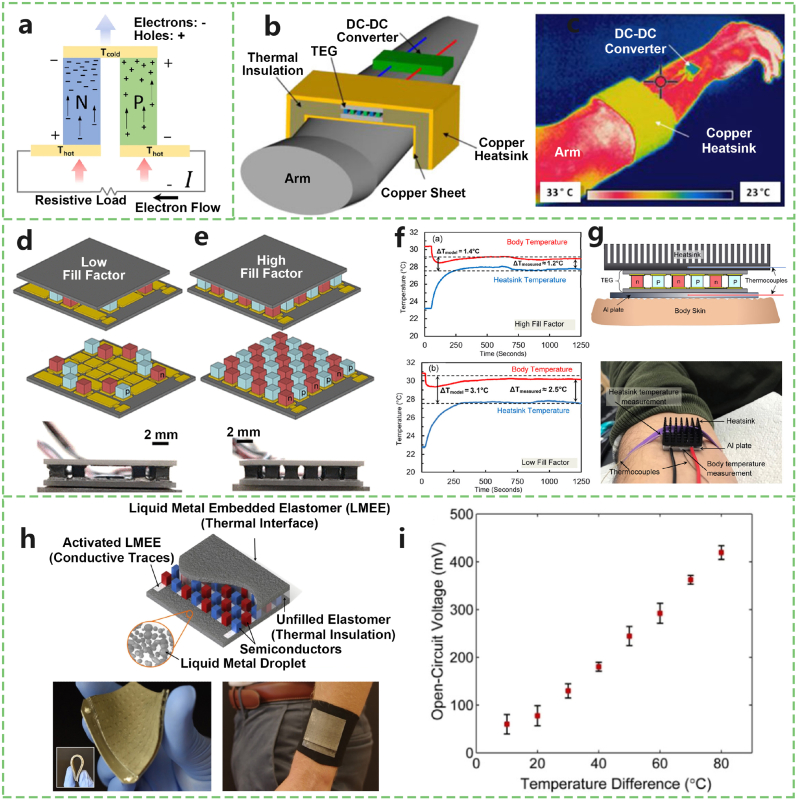
Wearable devices powered by a body thermal gradient. a) Schematic of a TEG based on the Seebeck effect. b) Structural schematic of a low-profile wearable TEG device. c) Infrared thermal image of a copper component coated with high-emissivity material. d) Thermoelectric module design with a low fill factor (12 %) and e) a high fill factor (36 %) for enhanced body heat harvesting. f) Experimental measurements of the temperature on the hot (skin) side and the cold (heatsink) side for both high (top) and low (bottom) fill factor modules. g) Schematic and photographic representation of the temperature measurement setup involving thermocouples and a metal heatsink. h) Schematic diagram of a stretchable wearable TEG using a thermally conductive LMEE as the thermal interface on both surfaces. i) Open-circuit voltage output as a function of temperature difference across the LMEE-based TEG surface. Adapted from [62,63,64,65].

The exploration of body thermal gradient energy harvesting through TEGs represents a burgeoning field of research, particularly fascinating because of its potential to transform the omnipresent but often disregarded heat produced by the human body into a valuable source of power for electronic devices. This form of energy harvesting capitalizes on the Seebeck effect, where a temperature differential between two materials generates an electric voltage (Fig. 5a). By leveraging the body's consistent thermal output, researchers aim to sustainably power wearable technologies and implantable medical devices, presenting a promising solution for the energy needs of small-scale electronics.

Studies have demonstrated various applications and efficiencies of TEGs in harnessing body heat. For instance, Nedelcu et al. [66] presented a case study showing the potential of generating approximately 1 W of power with a mean temperature difference of 50 °C between the hot and cold sources of a TEG, highlighting the scalability of such systems by adding more modules. Similarly, Settaluri and colleagues [62] focused on a thin TEG system specifically designed for body energy harvesting, illustrating the adaptability of TEGs to wearable formats (Fig. 5b and c). Notably, Jo et al. [67] managed to generate an output power of 2.1 μW with a temperature difference of 19 K between the human body and ambient air, underscoring the potential of TEGs in low-power applications. However, the efficiency of these systems often depends on the magnitude of the temperature differential they can exploit. Techniques such as radiative cooling and integration with phase change materials have been proposed to maximize the temperature gradient and thus the energy output from these generators. For example, a study demonstrated an innovative approach in which simultaneous radiative cooling and solar heating significantly increased thermoelectric voltage production by increasing the temperature gradient [68].

A study by Smith and Johnston [69] used wearable devices to evaluate the potential for harvesting electrical energy from body heat. By placing TEGs on the arms of three subjects, they achieved an average continuous maximum power output of 22.9 μW, with a power density of 1.43 μW/cm^2^. When the TEGs were initially placed on the skin, a significant thermal gradient was created, allowing the TEGs to provide sufficient voltage on a matched load to enable the cold start of state-of-the-art DC‒DC boost converters. The results indicate that centimeter-scale TEGs have enough power density and voltage output to power battery-less wearable sensor devices by harvesting body heat.

Nozariasbmarz et al. [63] developed a low fill factor TEG module designed for wearable devices. The low fill factor refers to the proportion of the TEG base area occupied by the legs (Fig. 5d– g). This design offers a larger temperature difference, enhancing power generation efficiency while reducing material usage, cost, and weight. However, modules with a fill factor less than 15 % lack mechanical robustness, so commercial modules typically use a fill factor of 25–50 %. Their manufactured module maintains robustness while significantly improving performance, with the output power increased by approximately 80 % compared with that of commercial designs and a power density reaching 35 μW/cm^2^. This enhancement is achieved by using two-thirds less thermoelectric material, demonstrating its potential as a high-specific-power-density stable power source, driving the development of wearable devices.

The primary allure of harnessing body thermal gradients lies in its sustainability and the ubiquity of the energy source—the human body. TEGs offer a silent, maintenance-free method to generate electricity, potentially eliminating the need for batteries in certain wearable and medical devices. This could lead to lighter, more comfortable devices with significantly longer lifespans, contributing to both user convenience and environmental sustainability. Despite these advantages, several challenges persist. The efficiency of TEGs, particularly at the relatively low temperature differentials presented by the human body, remains modest. This limitation necessitates the optimization of TEG materials and structures to increase their power output for practical applications. Furthermore, the integration of TEGs into wearable devices must be approached with careful consideration of comfort and aesthetics, ensuring that the addition of such technology does not impede the device's functionality or user acceptability. While body thermal gradient energy harvesting through thermoelectric generators offers an enticing pathway to power the next generation of electronic devices, substantial research and development are needed to overcome its current limitations. Advancements in materials science, coupled with innovative design strategies, hold the key to unlocking the full potential of this sustainable energy source.

With respect to materials and structures, **Mason Zadan et al.** [64] developed a soft-matter TEG using liquid-metal elastomer (LMEE) composites with Bi2Te3 semiconductors, maintaining performance under strains over 50 % and generating power from various temperature gradients (Fig. 5h and i). **Jiahui Li et al.** [70] created stretchable n-type thermoelectric fibres using Ti3C2Tx MXene nanoflakes and polyurethane composites, achieving a textile thermoelectric generator with a voltage of 3.6 mV from the temperature gradient between the skin and the environment. **Yeongju Jung et al.** [71] introduced a stretchable and breathable thermoelectric skin with both radiative cooling (RC) and solar heating (SH) functions, achieving a maximum power output of 5.73 μW cm^−2^ in RC mode and 18.59 μW cm^−2^ in SH mode. A notable study by Jung et al. [72] demonstrated a soft, stretchable thermoelectric skin (TES) capable of dual-mode operation: harvesting energy from underwater thermal gradients and actively regulating body temperature via the Peltier effect. The device utilized a serpentine Cu electrode array embedded in a thermally conductive elastomer (Ag-Ecoflex), achieving 230 % stretchability and generating up to 4.8 μW/cm^2^ under a 14 K temperature gradient. This innovation highlights the potential of WTEGs in extreme environments, such as underwater diving, where energy autonomy and thermal regulation are critical.

### 3.5 Voluntary movement

**Fig. 6:**
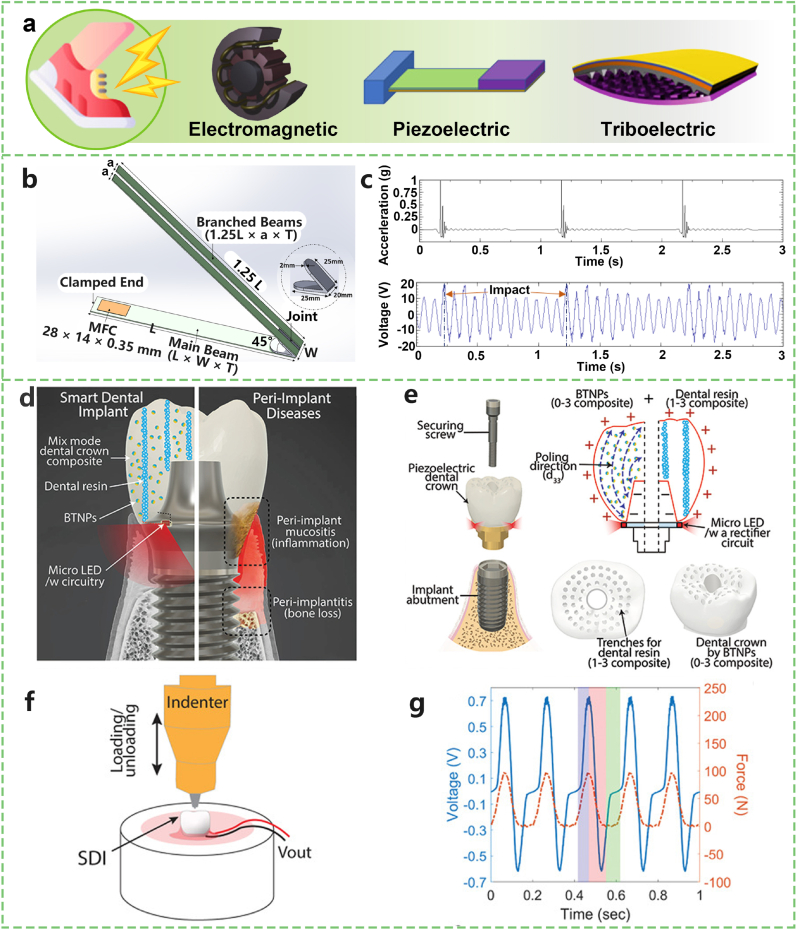
Wearable or implantable devices powered by human voluntary movement. a) Common strategies for harvesting energy from human voluntary movement, including electromagnetic, piezoelectric, and triboelectric mechanisms. b) Structural design of the BBBH. c) Measured base acceleration and corresponding voltage output of the BBBH under 1 Hz vibration frequency and 1 g acceleration. d) Conceptual overview of the SDI system designed for peri-implant disease detection. e) Schematic of the SDI assembly featuring a screw-retained dental implant structure with a two-phase composite dental crown, integrated microelectronics, and micro-LEDs. f) Experimental setup for characterizing the SDI under cyclic loading (chewing motion). g) Voltage outputs and applied force over time recorded from the SDI during a chewing simulation in the absence of a circuit. Adapted from Refs. [73,74,75].

Harnessing energy from voluntary motion offers an innovative approach to powering wearable electronics and implantable medical devices, making strides towards sustainability in the realm of personal electronics. Utilizing electromagnetic, piezoelectric, and triboelectric strategies (Fig. 6a), kinetic energy can be transformed from everyday activities—such as walking, running, and even subtle movements such as arm swings—into electrical energy. The principle behind this technique is to capture the energy generated by movements that would otherwise be wasted and convert it into a useable form of power.

Studies have explored various aspects of kinetic energy harvesting from voluntary motion. For instance, Blokhina et al. [76] delved into patterns and self-similarity of acceleration time series from human movements, emphasizing how activities such as running or walking are particularly suitable for near-limit kinetic energy harvesters. In another innovative approach, Wang et al. [77] demonstrated that tristable energy harvesters outperform linear harvesters in harvesting vibration energy from human walking or running, showing the potential for efficiency improvements in energy capture from human activities. Despite promising advancements, challenges persist in optimizing the energy harvesting process. Low frequencies and irregular motions characteristic of human movement pose difficulties in maximizing energy extraction [78]. To address these challenges, researchers have employed techniques such as motion tracking systems, pressure sensors, and electromyography (EMG) to analyse human locomotion in greater detail, aiming to increase the efficiency of energy harvesters [79]. Iresha Erangani Piyarathna et al. [73] proposed a novel bent branched beam piezoelectric energy harvester (BBBH) to improve energy generation from ultralow-frequency vibration sources, such as voluntary motion (Fig. 6b and c). Through numerical and experimental studies, the BBBH demonstrated significant improvements in operating bandwidth, voltage, and power generation. Tests revealed that the BBBH achieved three close resonance peaks in the 1 Hz–10 Hz range, with a maximum output voltage of 62 V, and significantly reduced idle time. Compared with traditional designs, the performance enhancement of the BBBH does not require additional components, making it suitable as a standalone energy provider for low-power devices.

Piezoelectric nanogenerators (PENGs) and triboelectric nanogenerators (TENGs) are utilized to convert kinetic energy from voluntary motion into electrical energy. For example, Park et al. [74] proposed a smart dental implant (SDI) system that collects energy from chewing and tooth-brushing motions through a piezoelectric dental crown (Fig. 6d and e). The energy is stored in a 47 μF capacitor, with a voltage output ranging from 0.4 to 1.3 V (Fig. 6f and g), depending on the motion. The mechanical strength of this system is comparable to that reported in other studies [[80], [81], [82], [83],^84^], confirming its suitability as a dental implant material.

The principal advantage of harvesting energy from voluntary motion lies in its potential to provide a continuous, renewable power source for low-power devices, reducing dependency on batteries and thereby minimizing environmental impact [85]. This technology could revolutionize the way wearable devices are powered, leading to advancements in healthcare monitoring, fitness tracking, and even in the development of self-sustaining medical implants. However, the effectiveness of kinetic energy harvesters is often limited by the variable nature of human movement, which can result in inconsistent energy outputs [86]. The current efficiency of these devices may not yet meet the power requirements of many electronic devices, necessitating further research and development to increase their performance. Additionally, integrating these energy harvesters into wearable devices without compromising comfort or hindering movement remains a significant engineering challenge. While kinetic energy harvesting from voluntary motion presents an exciting frontier in sustainable energy for wearable and implantable devices, realizing its full potential requires overcoming significant technical hurdles. Advances in materials science, biomechanics, and energy conversion technologies are crucial for optimizing the efficiency and practicality of these systems [87], promising a future where electronics can be powered seamlessly by the very movements they are designed to track or augment.

### 3.6 Glucose oxidization

The innovative realm of glucose oxidization in biofuel cells (BFCs) has revealed a spectrum of possibilities for energy harvesting, especially for powering biomedical and wearable devices. This technique involves the biochemical conversion of glucose, a prevalent and renewable energy source within the human body, into electrical energy, harnessing the catalytic ability of enzymes such as glucose oxidase. Here, some pivotal studies that illuminate the potential and challenges of this promising technology are more closely reviewed.

**Fig. 7:**
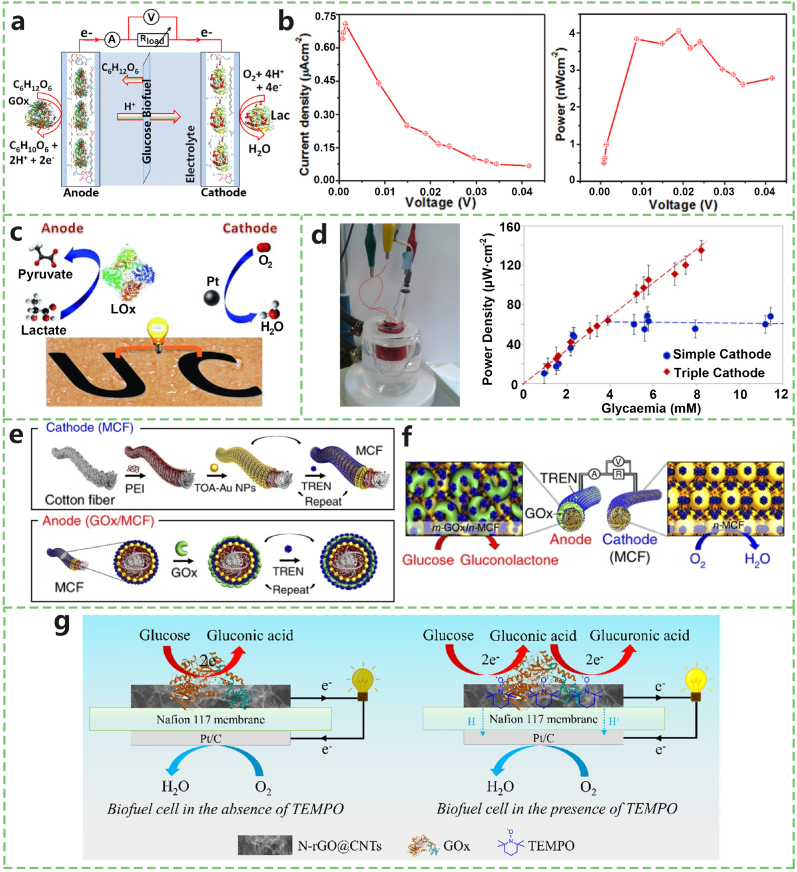
Implantable devices powered by glucose oxidation. a) Schematic illustration of an enzymatic BFC operating via glucose oxidation, with a GOx/reduced graphene oxide (rGO) bioanode and a laccase/rGO biocathode. b) Polarization and power density curves of the BFC measured in oxygen-saturated 0.01 M PBS containing 40 mM glucose. c) Flexible BFC integrated with lactate oxidase on the anode and platinum on the cathode. d) Experimental setup of the BFC tested in whole human blood samples. e) Power output as a function of glycemia level in human blood, comparing simple versus triple cathode configurations. f) Schematic of the small-molecule ligand-induced layer-by-layer assembly process for the MCF-based BFC electrodes. g) Redox mechanism of the MCF-BFC system, showing glucose oxidation at the anode and oxygen reduction at the cathode. h) Schematic diagram of a glucose/air enzymatic BFC comprising a HEOC anode and an air-breathing platinum cathode. Adapted from Refs. [89,90,93,94] [95].

Gazizova et al. [88] proposed an ingenious multisource energy harvesting system that synergizes an enzymatic BFC with a thermoelectric generator (TEG). This hybrid system is designed to capitalize on human perspiration and body heat to generate electricity, offering a compelling solution for the sustained operation of wearable devices. This holistic approach underscores the viability of integrating diverse energy harvesting methods to enhance reliability and output. In the quest for efficient and biocompatible electrode materials, Kabir et al. [89] explored the use of filter paper as a cost-effective substrate in enzymatic biofuel cells (Fig. 7a and b). This investigation not only emphasized the importance of accessible materials but also highlighted advancements in mediator-free glucose-powered cells, paving the way for broader applications in the biomedical, agrofood, and environmental sectors. Jia et al. [90] introduced a novel application of epidermal biofuel cells by generating electrical power from human perspiration via temporary transfer tattoos (Fig. 7c). This study highlights the potential of epidermal biofuel cells in creating noninvasive, wearable power sources, revolutionizing how energy can be harvested from the human body. In a critical analysis of the hypoglycemic risks associated with implantable glucose biofuel cells, especially in type 2 diabetic patients, Tang Foon and Rocke [91] employed glucose‒insulin simulation models. Their work emphasized the necessity of considering patient safety and metabolic impacts in the development of biofuel cells for medical applications. S and Goel [92] highlighted the suitability of biofuel cells as optimal power sources for wearable bioelectronics, emphasizing the direct generation of energy from the physiological environment. This perspective highlights the importance of seamlessly integrating biofuel cells with the biological milieu to provide sustainable energy solutions for wearable technologies. Marine Cadet et al. [93] demonstrated that by finely tuning the anodic and cathodic bioelectrocatalysts, the power density of enzymatic BFCs is directly proportional to the glucose level in human blood (Fig. 7d). They achieved a maximum power density of 129 μW/cm^2^ at 0.38 V vs. Ag/AgCl with 8.22 mM glucose, which is the highest power density reported to date in human blood. At 0.47 V, the power density was 21 times greater than previously reported values. BFCs offer biocompatibility and mild operating conditions, but their application is limited by relatively low output power.

In terms of innovative materials and techniques, Cheong Hoon Kwon et al. [94] demonstrated ultrahigh-power direct electron transfer biofuel cells (DET-BFCs) using glucose oxidase-coated metallic cotton fibres (MCF) as electrodes in hybrid BFCs (Fig. 7e and f). By employing small-molecule linker-derived metal nanoparticles and enzyme multilayers, they significantly enhanced electrical communication between electrochemically active components and maximized the electrocatalytic activity of highly porous cotton fibres. Increasing the number of TOA-Au NP/TREN bilayers further improved the electrical conductivity and electrocatalytic surface area of the MCF-BFCs, resulting in an areal power density of 3.7 mW/cm^2^, indicating the best reported performance. This method optimizes both the electrical communication and electrochemical performance of the electrodes. Itthipon Jeerapan et al. [96] introduced a glucose/oxygen-driven BFC that operates efficiently under oxygen-deficient conditions. This novel BFC utilizes an oxygen-rich polychlorotrifluoroethylene (PCTFE)/ionic liquid (IL)-based carbon/Pt-coated cathode as an internal source of oxygen, ensuring effective oxygen reduction reactions. In oxygen-free media, the cell can maintain over 90 % of its initial power during prolonged operation. The high oxygen solubility of the PCTFE fluorocarbon binder facilitates oxygen reduction reactions under severe oxygen-deficit conditions. The superior oxygen-storage capability of the composite cathode enables the BFC to operate continuously under fluctuating oxygen levels and anaerobic conditions. This oxygen-rich cathode strategy not only enhances the performance of bioenergy conversion systems but also offers new opportunities for advanced biosensing applications in anaerobic systems. Gangyong Li et al. [95] proposed and utilized a hybrid enzymatic and organic catalyst cascade system to enhance glucose oxidation and fabricate glucose/air enzymatic biofuel cells (Fig. 7g). Their system combines the organic oxidation catalyst 2,2,6,6-tetramethyl-1-piperidine N-oxyl (TEMPO) with the enzyme glucose oxidase (GOx). TEMPO mediates electron transfer between GOx and the electrode, enabling efficient 4e^−^ oxidation of glucose under neutral conditions. The resulting glucose/air biofuel cell with a TEMPO-based high-electrocatalytic oxygen reduction (HEOC) anode and an air-breathing Pt cathode achieved a maximum power density of 38.1 μW cm^−2^ and a short-circuit current of 651.4 μA cm^−2^, demonstrating improved energy efficiency. They used analytical-grade reagents, including GOx, Nafion 117 membranes, TEMPO, and other materials from commercial sources. This approach highlights a new strategy for glucose oxidation under mild conditions, which significantly enhances the catalytic efficiency through the synergistic action of enzymatic and organic catalysts. Phan Gia Le and Moon Il Kim [97] proposed noble metal-based nanozyme biofuel cells (NBFCs) to construct implantable devices that generate power from real biological fluids. For example, an NBFC implanted in humans produced 104 μW of power at normal glucose levels and was used for a pacemaker. However, the high cost and toxicity of noble metal-based nanozymes limit their practical application. In contrast, metal oxide-based nanozymes offer cost advantages.

Ho et al. [98] demonstrated a metal oxide-based NBFC using CoMn_2_O_4_/carbon as an anode catalyst, achieving a power output of 2.372 mW/cm^2^, comparable to that of commercial Pt/carbon-based systems. Another NBFC using Co_3_O_4_ and nitrogen-iron-doped activated carbon showed a power output of 12.81 μW/cm^2^, although the performance decreased at high glucose concentrations. Slaughter et al. [99] used ZnO nanosol and Pt to construct an NBFC, which displayed a power output of 16.2 μW/cm^2^ and an open-circuit voltage of 0.84 V. The electronic structure of ZnO helps explain the glucose-to-electricity conversion mechanism.

The primary allure of glucose oxidization in biofuel cells lies in its utilization of a readily available, renewable resource, glucose. This approach promises to serve as a sustainable power source for wearable and implantable devices, potentially reducing their dependency on conventional batteries and minimizing their environmental impact. Key challenges include the long-term stability and activity of enzymatic catalysts, which can degrade over time, and the generally low power output, which may not meet the demands of all medical devices or wearable technologies. Additionally, ensuring biocompatibility and safety for implantable applications is crucial. To overcome these challenges, ongoing research has focused on enzyme stability, power output enhancement, and biocompatibility. Advancements in materials science, enzyme engineering, and nanotechnology are essential for unlocking the full potential of glucose oxidization in biofuel cells for a wide range of applications.

Glucose oxidization in biofuel cells represents a promising and sustainable approach for powering medical and wearable devices, and addressing challenges related to enzyme stability, power output, and biocompatibility is critical. Continued research and development are needed to fully realize the potential of this technology.

## 4 Energy management and storage

In the realm of advancing sustainable power solutions for medical devices, a critical obstacle lies in the effective management and storage of energy. Traditional batteries, while fundamental, exhibit intrinsic limitations such as finite lifetimes, the requirement for periodic replacement or recharging, and potential hazards such as leakage or malfunction. The energy harvested from ambient sources often displays fluctuations and unpredictability, necessitating robust systems to ensure the stable operation of medical devices. In response to these challenges, researchers are exploring alternative energy storage options, notably, supercapacitors and biofuel cells.

While batteries are still commonly used in medical devices, advancements are being made to improve their performance and reliability. For example, researchers are developing microbatteries that are smaller, lighter, and more energy dense than traditional batteries are, making them more suitable for use in implantable devices [100]. Additionally, efforts are being made to develop biodegradable batteries that can safely disintegrate within the body after fulfilling their purpose [101].

Supercapacitors, also known as ultracapacitors, offer several advantages over traditional batteries, including higher power density, longer cycle life, and faster charging times. They are particularly well suited for applications that require short bursts of high power, such as those encountered in many medical devices. Advances in materials and fabrication techniques have led to the development of supercapacitors with improved energy density and performance, making them more competitive with batteries for use in sustainable power systems [102].

Biofuel cells represent a promising alternative to traditional batteries and supercapacitors for sustainable power systems. These devices harness energy from biochemical reactions within the body, such as the oxidation of glucose or oxygen reduction, to produce electricity. Biofuel cells offer several advantages, including high energy density, long operational lifetime, and compatibility with the human body. Researchers are actively developing biofuel cells with improved performance, efficiency, and biocompatibility, making them a viable option for powering implantable medical devices [103].

Hybrid systems that combine multiple energy storage technologies, such as supercapacitors and microbatteries, are also being explored to take advantage of the strengths of each technology [104]. These systems can provide both high power density for short bursts of energy and high energy density for sustained operation.

## 5 Discussion

### 5.1 Summary

The landscape of power sources for IMDs and wearable technologies is undergoing a revolutionary transformation with the adoption of innovative energy harvesting techniques. This evolution encompasses a diverse array of methods, each with a distinct mechanism and potential applications. Biocompatibility and hemocompatibility considerations are paramount in the development of these implantable devices, ensuring their safe and effective integration within the human body.

Electromagnetic energy harvesting stands out for its ability to enable simultaneous power transfer and data exchange, offering a promising pathway for devices such as smart contact lenses while maintaining a high level of biocompatibility. Similarly, US-WPT harnesses sound or vibration waves, facilitating deeper penetration and minimal interference with internal electronics, ensuring compatibility with the body's physiological environment. Tissue motion and heartbeat energy harvesting leverage the body's constant movements, providing a continuous source of power with minimal impact on biological tissues and functions. Thermal energy differentials within the body are explored through TEGs, which utilize the Seebeck effect to convert body heat into electrical energy, with a focus on maintaining hemocompatibility. Voluntary motion harvesting uses everyday movement for energy generation, ensuring seamless integration with natural body functions. Additionally, glucose oxidation within biofuel cells presents a sustainable solution by converting biochemical energy from glucose into electrical power while ensuring biocompatibility and hemocompatibility.

Each of these methods presents unique advantages, ranging from deep tissue penetration and noninvasive energy transfer to harnessing inexhaustible biological processes for power generation. Table 2 highlights a critical trade-off: Biocompatible materials (e.g., polymers in TEGs) reduce power density by 40–60 % compared to inorganic alternatives. Conversely, high-output electromagnetic harvesters risk tissue heating. Future breakthroughs demand material innovations such as self-annealing piezoelectric polymers or enzyme-stabilized metamaterials to resolve this dichotomy. The consideration of biocompatibility and hemocompatibility is fundamental in the design and implementation of these energy harvesting techniques for implantable medical devices and wearable technologies, ensuring their efficacy and safety in clinical applications.

**Table 2:** Comparison of power strategies for IMDs and wearable technologies.

Energy Harvesting Technique	Power Source	Power Transfer Efficiency	Power Output (Watts)	PTE Improvement Strategies	Biocompatibility	Long-Term Health Effects	Applicability to Specific Use Cases
**Electromagnetic Energy Harvesting**	Ambient electromagnetic	Medium-high	5–10 mW	Utilize metamaterials for enhanced antenna efficiency, employ metasurfaces for improved energy absorption and conversion	Concerns over electromagnetic exposure, biocompatibility	Long-term health effects not well-studied	Suitable for smart contact lenses, IoT medical devices
**US-WPT**	Ultrasonic waves	Medium-high	10–20 mW	Implement metamaterial structures for enhanced ultrasound focusing, optimize transducer designs for increased efficiency	Biocompatible, minimal tissue interference	Potential for tissue heating, long-term safety	Ideal for deep tissue penetration, noninvasive energy transfer
**Spontaneous organ activity**	Mechanical tissue movements	Low-medium	1–5 mW	Develop advanced energy conversion mechanisms, optimize materials for enhanced energy capture	Generally, biocompatible, harnesses body motion	Potential for tissue wear, long-term reliability	Well-suited for wearable sensors, pacemakers, orthopedic implants
**TEGs**	Body heat differentials	Medium	5–15 mW	Enhance thermoelectric materials using metamaterial structures, optimize thermal interface materials for improved heat transfer	Biocompatible materials, internal thermal management	Thermal effects on tissues, long-term safety concerns	Applicable for body-heat-powered wearables, implantable devices
**Voluntary movement**	Human movement	Medium-high	3–10 mW	Optimize energy capture mechanisms, enhance materials for improved kinetic energy conversion, minimize power loss	Biocompatible, uses natural body motion for power	Potential for tissue strain, long-term reliability	Effective for wearable fitness trackers, activity monitors, prosthetics
**Glucose-based Biofuel Cells**	Glucose oxidation	Low-medium	1–8 mW	Develop biocompatible electrode materials, enhance catalytic efficiency, improve glucose transport mechanisms	Potential for biocompatibility, sustainable power source	Issues related to enzymatic stability, immune response	Suitable for glucose monitoring devices, long-term implantable sensors

Within the scope of the technologies discussed in this paper, a crucial distinction arises between implantable devices and skin-attachable devices, each demanding unique considerations. Implantable devices, intended for placement inside the body, necessitate materials that are biocompatible to mitigate the risk of rejection or adverse reactions. These devices typically require surgical implantation procedures, precise engineering for enduring reliability, and power sources that are both sustainable and safe within the body's internal environment. Compliance with stringent biocompatibility and safety standards is paramount to ensure harmonious integration with bodily tissues and functions, alongside considerations of longevity and resilience to physiological conditions.

On the other hand, skin-attachable devices are designed for temporary adhesion to the skin's surface, necessitating materials that are flexible, breathable, and comfortable for extended wear. Their construction should enable secure attachment to the skin without causing irritation or discomfort, with a focus on lightweight, unobtrusive design for user comfort. Ease of application and removal, along with factors such as adhesion strength, skin compatibility, and the ability to efficiently collect data or provide feedback, are key considerations for skin-attachable devices. Understanding and addressing the distinct requirements of implantable and skin-attachable devices are pivotal in tailoring their design, materials, and functionality to meet their specific purposes effectively.

The comparison Table 3 provides a detailed overview of the manufacturing aspects for each energy harvesting device type, encompassing manufacturing cost, time requirements, components involved, and labor details. These parameters play a critical role in assessing the feasibility and practicality of implementing these technologies in medical devices and wearables. However, despite the promising prospects of these energy harvesting methods, challenges exist that need to be addressed.

**Table 3:** The manufacturing of different power strategies for IMDs and wearable technologies.

Energy Harvesting Technique	Applications	Manufacturing Cost	Time	Components	Labor
**Electromagnetic Energy Harvesting**	Pedestrian pavements, rail systems, IoT sensors	Moderate (50–500 per unit). Costs depend on magnet/coil quality and scale	Weeks (assembly of coils, magnets, and mechanical components)	Coils, permanent magnets (e.g., Halbach arrays), flywheels, gears, PCB for power management	Skilled labor for precision assembly; automated winding for coils reduces costs
**US-WPT**	Implantable medical devices	High (100–1000 per unit). AlN PMUT arrays and impedance-matching circuits are expensive	Months (CMOS-compatible fabrication for AlN thin films).	AlN piezoelectric transducers, voltage multipliers, power management ICs, capacitors	Specialized (cleanroom fabrication for MEMS; PCB assembly for circuits).
**Spontaneous organ activity**	Pacemakers, biosensors	Very high ($1000+). Biocompatible materials (e.g., Pt/rGO) and sterile packaging add costs	Months to years (clinical testing dominates timelines).	Abiotic catalysts (e.g., Fe-Co/KB), glucose/O_2_ electrodes, biocompatible casings	Highly specialized (electrochemical engineers, medical device regulators)
**TEGs**	Wearables, health monitors	Low to moderate (20–200). Flexible Bi_2_Te_3_/PVDF composites reduce costs	Days to weeks (drop-casting or screen-printing methods)	Thermoelectric films (e.g., Bi_2_Te_3_), flexible substrates (PET), heat sinks	Semi-skilled (printing processes); R&D-intensive for material optimization
**Voluntary movement**	Footstep harvesting, smart floors	Low (10–100). PZT materials are cheap but brittle; polymers (PVDF) cost more	Days (simple designs) to months (complex mechanical linkages)	Piezoelectric stacks (PZT), triboelectric layers (e.g., PDMS), springs/gears	Low to moderate (assembly-line production for tiles; skilled for precision devices)
**Glucose-based Biofuel Cells**	Implantable sensors	High ($500+). Noble metal catalysts (e.g., Pt) and sterilization are costly	Months (enzyme-based cells require stability testing)	Anodes (glucose-oxidizing catalysts), cathodes (ORR catalysts), membranes	Highly specialized (biocompatibility testing, electrochemistry expertise)

Electromagnetic energy harvesting faces hurdles such as weak coupling due to misalignment and potential health concerns from prolonged exposure to electromagnetic fields. To enhance this technology, advanced alignment techniques and improved safety measures regarding electromagnetic fields are essential for its development. Ultrasound Wireless Power Transfer (US-WPT) encounters challenges related to coupling efficiency and potential health implications from extended exposure to ultrasound waves such as standing wave formation and transducer misalignment. The future of US-WPT lies in advancements in transducer technology to improve coupling efficiency and in optimizing power transmission methods to minimize health risks associated with its usage, like beam steering through phased array systems or the use of miniaturized implantable receivers. Energy harvesting from spontaneous organ activity entails challenges in ensuring consistent energy extraction from varying organ movements and optimizing energy conversion efficiency. To overcome these obstacles, advancements in sensor technology for precise energy capture and innovative designs to enhance energy conversion efficiency are crucial for the evolution of this technology. Thermoelectric Generators (TEGs) face challenges due to modest efficiency at low temperature differentials and the necessity to ensure the long-term stability of TEG materials. The future of TEGs involves research into advanced materials and the optimization of designs to achieve increased efficiency and stability in energy conversion.

Voluntary movement energy harvesting encounters issues related to the variability in energy output based on individual activity levels and the need for precise calibration to ensure accurate energy capture. Advancements in motion sensor technology and the development of sophisticated calibration methods are key for enhancing energy harvesting from voluntary movements. Glucose-Based Biofuel Cells strive to maintain the long-term stability of enzymes and optimize the efficiency of glucose oxidation. Research into enzyme stabilization techniques and advancements in biofuel cell materials are crucial for enhancing the efficiency and stability of biofuel cells in energy harvesting applications.

What's more, there are safety considerations in extending battery life for IMDs and wearable technologies, since wiring and electronic components may wear out before the battery does, creating unexpected failure modes. Complete lifetime of embedded devices including wires should be considered at the same time. Addressing the challenges necessitates a multidisciplinary approach integrating advancements in materials science, biomechanics, biochemistry, and electrical engineering. By overcoming these obstacles, the future of energy harvesting devices for medical applications holds great promise for sustainable, efficient, and biocompatible power solutions in healthcare.

While glucose biofuel cells offer superior biocompatibility, their manufacturing costs exceed $500/unit due to noble-metal catalysts—limiting scalability. Conversely, voluntary-motion harvesters are cost-effective but suffer from >30 % performance decay after 6 months in vivo. Hybrid manufacturing approaches (e.g., 3D-printed bioreactors with embedded piezoelectrics) could bridge this gap.

### 5.2 Regulatory and ethical consideration

As sustainable power technologies for medical devices continue to advance, it is crucial to consider the regulatory and ethical implications of these innovations. Regulatory bodies such as the Food and Drug Administration (FDA) in the United States [105] and the European Medicines Agency (EMA) in Europe [106] play pivotal roles in ensuring the safety and efficacy of these devices before they reach the market. These agencies typically require rigorous premarket testing, clinical trials, and adherence to specific guidelines regarding device design, materials used, and data privacy [107].

Ethically, the development and deployment of sustainable medical devices have raised several considerations. For example, ensuring patient consent and autonomy in the use of these devices is essential [108]. Patients should be fully informed about the potential risks and benefits associated with the technology. Additionally, there are concerns regarding data privacy and security, especially as these devices may be continuously collecting and transmitting personal health data. Researchers and manufacturers must implement robust cybersecurity measures to protect sensitive information [109].

### 5.3 Future prospective

The future of energy harvesting for IMDs and wearable technologies hinges on overcoming these obstacles through innovations in nanotechnology, the development of smart energy management systems, and the exploration of hybrid systems that combine the strengths of multiple energy harvesting techniques. This integrated and adaptable approach holds the promise of unlocking new possibilities for powering the next generation of medical and wearable devices, representing a significant stride toward sustainable and efficient energy solutions, for example, in high-throughput sensing in biology, organ-wide selective drug delivery, and deep tissue stimulation [110,111]. Recently, a study by Zain Ul Abdin et al. [112] reported an efficient and reliable WPT system for the surveillance of abdominal aortic aneurysms, a serious condition that requires continuous monitoring. The researchers designed a stent rectenna system, which combines a stent with a rectifying antenna, to act as an implantable receiver for WPT, indicating the feasibility of using this approach for powering implantable medical devices. By embracing these advancements and addressing existing challenges, the field of energy harvesting is poised to revolutionize the way we power and interact with medical and wearable technologies, paving the way for a more sustainable and technologically advanced future [113].

The integration of artificial intelligence (AI) into medical electronics presents an unprecedented opportunity to revolutionize implantable and wearable device technology. For example, AI-driven energy management systems have demonstrated exceptional potential in prolonging device lifespans by leveraging real-time data analytics to predict and optimize power usage. Adaptive algorithms in AI-enabled pacemakers analyse patient-specific heart rhythms to dynamically regulate energy consumption [114,115]. Similarly, wearable devices employing AI can adjust functionality on the basis of activity patterns, significantly reducing energy waste. These advancements extend to wireless power transfer systems, where intelligent control models optimize transmitter‒receiver alignment and compensate for variations in tissue density or movement [116,117].

Algorithm development further enhances energy harvesting efficiency by enabling wearable and implantable devices to adapt dynamically to user behavior and environmental conditions. Machine learning algorithms allow devices to predict energy utilization patterns, improving the sustainability and autonomy of these technologies. For example, adaptive energy management systems using machine learning can optimize power usage on the basis of user activity, extending the operational lifespan of self-sustaining devices [118,119]. Researchers from the University of Hong Kong have also developed a stretchable microelectronic platform based on organic electrochemical transistors, which significantly reduces energy consumption for AI-powered wearable sensing systems [115]. By integrating AI into energy harvesting systems, future wearable and implantable devices could achieve enhanced operational lifetimes and expanded functionality, paving the way for smarter, more sustainable medical technologies.

Beyond energy management, AI reshapes the design and manufacturing of medical devices. In the design phase, advanced simulation tools powered by AI can predict material performance, optimize device architecture, and ensure biocompatibility. The inherent contradictions exist between biocompatibility and power density. Achieving a balance between these essential factors poses a significant challenge in the development of energy harvesting technologies for biomedical applications. Biocompatibility requirements necessitate the use of materials that are safe for prolonged contact with biological tissues, ensuring minimal risk of adverse reactions or tissue damage. On the other hand, the need for adequate power density to sustain the operation of sophisticated medical devices complicates the selection of suitable power sources. Striking a harmonious equilibrium between these conflicting demands is essential to ensure the effectiveness and safety of sustainable power solutions in the context of implantable and wearable medical technologies. Addressing these technical bottlenecks through innovative design approaches and material selections using AI is crucial for advancing the field of sustainable energy harvesting for biomedical applications. This approach not only accelerates innovation but also fosters the creation of compact, durable devices. In manufacturing, AI-enhanced quality control systems detect microscopic defects via machine learning, whereas additive manufacturing techniques enable rapid prototyping and customization of medical devices, aligning with patient-specific requirements [120,121].

However, several challenges impede the full integration of AI in medical electronics. High computational demands often conflict with the constrained energy budgets of implantable devices, underscoring the need for energy-efficient AI models. Additionally, the reliance on large, high-quality datasets for training AI algorithms is complicated by patient variability and limited data availability in healthcare contexts [122]. Moreover, ensuring the reliability and safety of AI systems in life-critical scenarios remains a pressing concern, particularly for implantable devices where system failures could have severe consequences [123]. To address these challenges, future research must prioritize the development of lightweight, low-power AI models tailored for medical applications, as well as meeting safety requirements.

The future evolution of sustainable power solutions for medical devices hinges on resolving three fundamental dichotomies. First, the *biocompatibility-power density paradox* demands materials innovation: current high-output harvesters (e.g., PZT piezoelectrics, Bi_2_Te_3_ TEGs) risk cytotoxicity, while biocompatible alternatives sacrifice 40–60 % power density (Table 2). Multifunctional metamaterials—such as peptide-guided conductive networks within bioresorbable matrices—could decouple this trade-off by enabling efficient ionic transport without inflammatory responses. Second, *dynamic physiological coupling* remains unaddressed; organ motion variability (±35 %), metabolic fluctuations, and post-implantation tissue remodeling degrade energy capture. Closed-loop systems integrating biosensor feedback (e.g., impedance-tuned US-WPT, enzyme-modulated biofuel cells) and reinforcement learning algorithms could adapt harvesting priorities to real-time physiological states. Third, the *scalability-reality gap* persists: lab-scale demonstrations overlook manufacturing viability, as seen in noble-metal-dependent biofuel cells (>$500/unit) and low-yield MEMS fabrication (<15 %). Additive biofabrication of 3D-printed living electrodes with synthetic electroactive biofilms may enable scalable, self-regenerating power sources.

Beyond these challenges, transformative opportunities emerge. Neuromorphic processors could embed <10 μW adaptive control directly into harvesters, leveraging AI as a co-design partner to compensate for physiological noise. Cross-disciplinary synergies—such as bioinspired voltage-gated ion channels mimicking plant electrophysiology—may unlock ultra-low-power (<1 μW) sensing-actuation cycles. Ethically, >20-year implantable power sources necessitate "energy autonomy certification" frameworks and protocols for graceful performance degradation, ensuring patient safety transcends technical feasibility.

### 5.4 Conclusion

This review exposes a critical inflection point: sustainable medical devices cannot rely on incremental engineering but demand a paradigm shift toward bio-cooperative power systems. Such systems must function as synthetic organs—seamlessly integrating with host physiology through immune-evasive materials and dynamic energy negotiation (e.g., prioritizing motion harvesting during activity, thermal gradients during rest). Crucially, they should harness biological intelligence rather than brute-force extraction, leveraging endogenous signals like ATP gradients for enzymatic amplification. To achieve >15-year operational lifespans, self-healing architectures (e.g., polymer-embedded liquid metal circuits) and metabolic harmonization strategies must replace today's degradation-prone components.

Realizing this vision requires converging three domains: (1) Materials innovation via dynamic metamaterials that reconfigure to physiological conditions; (2) Computational paradigms employing embodied AI for real-time resource allocation; and (3) Regulatory evolution establishing lifespan certification standards. The broader implication extends beyond healthcare: such symbiosis between technology and living systems could pioneer sustainable interfaces for future bio-integrated technologies. Ultimately, the true measure of success lies not in harvested milliwatts but in achieving uninterrupted, autonomous operation that becomes indistinguishable from biological processes—a milestone demanding equal advances in fundamental science, clinical pragmatism, and ethical foresight.

## CRediT authorship contribution statement

**Ye Liang:** Writing – original draft. **Chi Zhang:** Writing – original draft. **Rubing Lin:** Writing – review & editing. **Junqing Lin:** Writing – review & editing, Supervision. **Jishizhan Chen:** Writing – review & editing, Writing – original draft, Conceptualization.

## Funding

This research received no specific grant from any funding agency in the public, commercial, or not-for-profit sectors.

## Declaration of competing interest

The authors declare that they have no known competing financial interests or personal relationships that could have appeared to influence the work reported in this paper.

## Data Availability

No data was used for the research described in the article.
